# CPAP modulation of sleep microstructure (N2-SSD and REM-AHI) as predictors of neurological recovery in ischemic stroke patients with moderate-to-severe OSA

**DOI:** 10.3389/fneur.2025.1605106

**Published:** 2025-08-26

**Authors:** Tianyu Jing, Liwen Xu, Shutong Sun, Wenyi Yu, Yixi Zheng, Gang Xu, Xinhao Shen, Tieyu Tang, Cheng Chu

**Affiliations:** ^1^Department of Neurology, The Affiliated Hospital of Yangzhou University, Yangzhou University, Yangzhou, China; ^2^School of Nursing and School of Public Health, Yangzhou University, Yangzhou, China; ^3^Intensive Care Unit, The Third People’s Hospital of Jiangyin City, Wuxi, China; ^4^Baoying County Yunxi People’s Hospital, Yangzhou, China

**Keywords:** continuous positive airway pressure, sleep spindles, ischemic stroke, REM-AHI, obstructive sleep apnea

## Abstract

**Background:**

There is a disease spectrum of ischemic stroke (IS) and obstructive sleep apnea (OSA), which are often comorbid in the same patient and consequently increases prognostic risk. Continuous positive airway pressure (CPAP) therapy is the primary treatment for stroke-related OSA; however, its positive effects on patient prognosis and the underlying mechanisms remain controversial.

**Objective:**

This study aimed to investigate the impact of CPAP therapy on the recovery of IS patients with moderate-to-severe OSA and to identify biomarkers significantly associated with prognosis to assess their predictive value for short-term neurological outcomes. The findings are expected to optimize treatment strategies and improve overall patient outcomes.

**Methods:**

A total of 141 patients with IS combined with moderate-to-severe OSA admitted to the Affiliated Hospital of Yangzhou University from October 2022 to August 2024 were enrolled. Patients were divided into a CPAP group (*n* = 68) and a control group (*n* = 73). Both groups received systematic treatment and were followed up until 1 month after the onset of stroke symptoms. The CPAP group initiated therapy within 48 h of stroke onset (ResMed AutoCPAP, pressure 4–20 cmH_2_O) for 14 days (adherence criterion: ≥4 h/day). Baseline data, sleep and stroke-related questionnaires, polysomnography (PSG) parameters, and sleep spindle characteristics were collected. Neurological functional outcomes were reassessed at the end of the follow-up period, and differences between the two groups were analyzed. Prognostic factors were identified using Spearman correlation analysis and ordered logistic regression.

**Results:**

Compared with those in the control group, patients in the CPAP group had lower modified Rankin scale (mRS) and National Institutes of Health Stroke Scale (NIHSS) scores after treatment (*p* < 0.05), while the Barthel index (BI) did not significantly differ. Spearman correlation analysis revealed that mRS scores were positively correlated with the apnea-hypopnea index (AHI), the AHI during the rapid eye movement stage (REM-AHI) and the AHI during the non-rapid eye movement stage (NREM-AHI) (all *p* < 0.05) and negatively correlated with the non-rapid eye movement stage 2 sleep spindle density (N2-SSD), the non-rapid eye movement stage 3 (N3) sleep percentage, and the mean pulse oxygen saturation (Mean SpO_2_) (all *p* < 0.05). Logistic regression revealed that N2-SSD, Mean SpO_2_, and REM-AHI were significant predictors of mRS scores (all *p* < 0.05).

**Conclusion:**

CPAP therapy enhances sleep microstructure and oxygenation parameters, which improves sleep quality. N2-SSD, REM-AHI, and Mean SpO_2_ are mechanistically linked to functional prognosis and CPAP exerts therapeutic effects through the modulation of these biomarkers. Early CPAP intervention targeting REM-AHI and N2-SSD demonstrates prognostic benefits, which suggests that sleep microstructure-specific metrics may serve as precision therapeutic targets.

## 1 Introduction

Ischemic stroke (IS) typically results from temporary or permanent blockage of cerebral blood vessels, which leads to brain tissue hypoxia, cell death, and a cascade of pathophysiological responses (1). Obstructive sleep apnea (OSA), a common sleep disorder, is characterized by frequent microarousals and recurrent oxyhemoglobin desaturation (2). These features are closely linked to both the incidence and prognosis of stroke. A bidirectional relationship exists between OSA and IS and is mediated by intermittent hypoxia and increased sympathetic activity (3). OSA contributes to cerebrovascular risk through mechanisms such as hemodynamic instability, vascular inflammation, intermittent hypoxia, and oxidative stress-induced free radicals (4). Moreover, OSA is frequently observed following IS. A meta-analysis on the incidence of OSA after stroke revealed that 72% of stroke patients had an apnea-hypopnea index (AHI) >5 events/h, and 29% had an AHI >30 events/h (5).

Patients with OSA complicated by IS (OSA-IS) face an elevated risk of developing additional complications, including recurrent stroke, extended hospitalization, diminished neurological function, and higher long-term all-cause mortality (4). Therefore, timely diagnosis and effective treatment are critical for patient management. Current guidelines recommend continuous positive airway pressure (CPAP) therapy as first-line treatment for moderate-to-severe cases when positional therapy is ineffective (6). However, there is substantial interindividual variability in CPAP efficacy, and its specific benefit for neurological recovery is not fully characterized (7).

Electroencephalography (EEG) can record electrophysiological changes related to the cerebral cortex. These findings provide rich biological information for the study of sleep structure and sleep quality in OSA patients. However, the EEG signals routinely collected by polysomnography (PSG) have not been fully utilized. Traditional metrics such as the AHI inadequately predict clinical outcomes, whereas PSG parameters and electrophysiological signals derived from EEGs remain underexplored. Thus, developing more sensitive biomarkers is imperative. This study aimed to evaluate the impact of initiating CPAP treatment within 48 h after the onset of ischemic stroke compared with standard treatment on neurological outcomes. After efficacy was determined, the correlation between PSG parameters and neurological outcomes was further explored to identify predictive biomarkers.

## 2 Methods

### 2.1 Study participants

This trial enrolled 141 patients with moderate-to-severe OSA-IS at the Affiliated Hospital of Yangzhou University from December 1, 2022 to August 31, 2024. The participants were divided into CPAP and control groups based on treatment preference. The inclusion criteria for patients were as follows: (1) Chinese adults aged ≥18 years; (2) met the diagnostic criteria for ischemic stroke per the Chinese Guidelines for Diagnosis and Treatment of Acute Ischemic Stroke (2023) (8) and moderate-to-severe OSA (AHI ≥15 events/h) per the Chinese Primary Care Guideline for Adult Obstructive Sleep Apnea (2018) (9). The diagnosis was confirmed by clinical history, neurological examination, and brain magnetic resonance imaging (MRI) with diffusion-weighted imaging (DWI) demonstrating acute ischemic lesions; (3) anatomically suitable for CPAP therapy (no craniofacial deformities). The exclusion criteria were as follows: (1) comorbid conditions affecting EEG signals (e.g., epilepsy, Parkinson’s disease, traumatic brain injury); (2) inadequate PSG data (total sleep time <5 h, <3 NREM-REM cycles, or unstable EEG signals); (3) contraindications to CPAP therapy; (4) use of neuroactive medications within 3 months; (5) delayed imaging confirmation of acute ischemic stroke (>24 h after symptom onset); (6) patients with aphasia or language comprehension deficits. The study protocol was approved by the Ethics Committee of Yangzhou University (Ethics 2023-YKL09) and conducted in accordance with the Declaration of Helsinki. Written informed consent was obtained from all participants and their families.

### 2.2 Information collection

#### 2.2.1 General information

Demographic information (age, sex distribution), body mass index (BMI), smoking and alcohol consumption history, and chronic medical conditions (hypertension, diabetes mellitus, coronary artery disease, atrial fibrillation) were collected. Additionally, imaging indices, such as CT and MRI images of the head, were included in this research.

#### 2.2.2 Assessment of sleep and cognitive function

This study uses the Pittsburgh Sleep Quality Index (PSQI), which is considered the gold standard in sleep research (10)_,_ to assess patients’ subjective sleep experience. The Epworth Sleepiness Scale (ESS) measures the degree of daytime sleepiness by assessing the likelihood of unintended dozing in daily situations (11). A total score exceeding 10 points indicates pathological excessive daytime sleepiness (EDS). The Rapid Eye Movement Sleep Behavior Disorder Screening Questionnaire (RBDSQ) includes 13 items designed to identify risk factors for Rapid Eye Movement Sleep Behavior Disorder (RBD) (12). The Hospital Anxiety and Depression Scale (HADS) is divided into two subscales: anxiety (HADS-A) and depression (HADS-D) (13). In this study, it is primarily used to assess participants’ emotional and cognitive symptoms, excluding somatic interference. The Subjective Cognitive Decline Questionnaire (SCD-Q9) is a self-assessment scale consisting of 9 items (14) and is used to detect early signs of subjective cognitive decline (SCD) in the memory and nonmemory domains. The Montreal Cognitive Assessment (MoCA) includes 12 tasks across seven cognitive domains to assess mild cognitive impairment (MCI) (15).

#### 2.2.3 Stroke evaluation metrics

The National Institutes of Health Stroke Scale (NIHSS), recognized as the gold standard for evaluating stroke severity (16), is used to assess neurological deficits across multiple domains, including consciousness, motor function (upper/lower limbs), ataxia, sensation, language, dysarthria, gaze, visual fields, facial palsy, and neglect. Scores were standardized to quantify the degree of neurological impairment. The Barthel Index (BI), a widely adopted tool for measuring post-stroke functional outcomes (17), evaluates activities of daily living through 10 items scored via a three-tier weighted system. The modified Rankin scale (mRS), the primary endpoint in acute stroke trials and quality improvement initiatives (18), categorizes functional recovery into seven grades (0–6), with higher scores indicating poorer recovery. This study uses this scale as the primary outcome measure. All assessments (NIHSS, BI, mRS) were assessed at admission and repeated at the end of the 1-month follow-up period.

### 2.3 Therapeutic protocol

Both groups received guideline-directed standard care for IS (19). The CPAP group additionally underwent CPAP therapy (ResMed AutoCPAP, pressure range: 4–20 cmH₂O) for ≥4 h nightly over 14 days. Initial manual pressure titration was performed by a certified respiratory therapist, who monitored patients overnight to optimize pressure settings based on tolerance. Data from the CPAP SD card, including residual AHI, total and daily usage time and air leakage, was recorded using the ResScan software post-treatment. After 14 days of CPAP treatment, participants in the CPAP group underwent repeat PSG under CPAP.

### 2.4 Polysomnography and sleep spindles analysis

All participants underwent overnight PSG (>7 h) in a light-attenuated, sound-controlled sleep laboratory using a SOMNOmedics GmbH system. Alcohol, caffeine, and stimulant/sedative medications were prohibited on the day of monitoring. Electroencephalogram (EEG), mandibular electromyography (EMG), electrooculography (EOG), nasal airflow, oro-nasal thermistor transducer, thoracic and abdominal movements, electrocardiogram, body position, oxygen saturation, snoring index, video, and other signals were recorded continuously throughout the night. The division of each sleep stage and sleep-related respiratory events was manually interpreted by a specialized sleep physician based on the standards of the American Academy of Sleep Medicine (AASM) (20). Nocturnal sleep-related parameters, including total sleep time (TST), sleep efficiency, wake-time after sleep onset (WASO), sleep onset latency, non-rapid eye movement stage 1 (N1)/non-rapid eye movement stage 2 (N2)/ non-rapid eye movement stage 3 (N3)/rapid eye movement stage (REM) percentage, the respiratory effort-related arousal index (RERAI), AHI, oxygen desaturation index (ODI), mean pulse oxygen saturation (Mean SpO_2_), and minimum pulse oxygen saturation (Min-SpO_2_), were continuously collected from the subjects.

The sleep spindle-related parameters were derived mainly from the central EEG (C4:M1), and the sleep spindle features were extracted as independent signals according to the algorithm established by Molle et al. (21), which used the Fourier transform to convert the EEG data into the frequency domain; then, 11–16 Hz bandpass filtering was performed. After that, signal smoothing was performed using a moving average method in 200 ms units, and finally, feature selection was performed on the basis of duration and amplitude (peak-to-trough amplitude). We collected the sleep spindle density (SSD), number of spindles, average spindle duration (in seconds), and maximum spindle amplitude (in μV) at the N2 and N3 stages. The SSD was calculated by determining the ratio of the number of spindle waves to the total duration of each N2 and N3 sleep stage.

### 2.5 Data analysis

All data were analyzed using SPSS Statistics (version 27.0; IBM Corp., Armonk, NY, USA). Continuous variables were assessed for normality using the Shapiro–Wilk test. Normally distributed data were presented as mean ± standard deviation (SD), while non-normally distributed data were expressed as median [interquartile range (IQR); Q1–Q3]. Between-group comparisons were analyzed using the independent *t*-test (for normally distributed data with homogeneity of variances) and the Mann–Whitney U test (for non-normally distributed data or unequal variances). Within-group comparisons were evaluated using the Wilcoxon signed-rank test for paired analyses. Categorical variables were expressed as frequencies or proportions, with group differences evaluated using the Chi-squared test. Correlation analyses were conducted using Spearman correlation analysis. Predictors of functional outcomes were identified through ordered logistic regression modeling with the mRS as the dependent variable. All statistical tests were two-sided, showing odds ratios (OR) with 95% confidence intervals (CI), and *p* < 0.05 was considered statistically significant.

## 3 Results

### 3.1 Comparison of baseline demographics, sleep-related questionnaires, and neurological function scores

**Figure 1:**
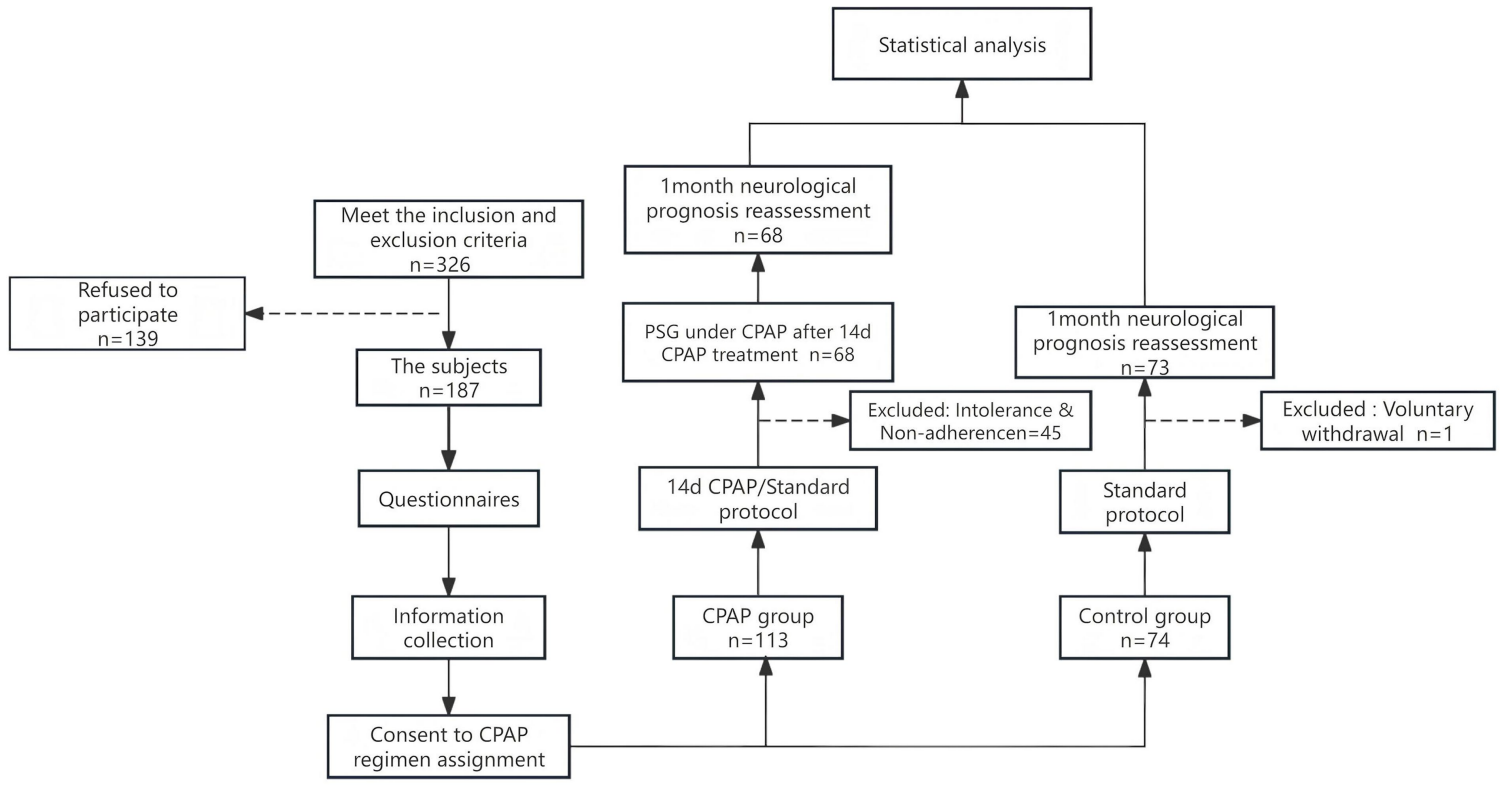
Research flowchart.

During the course of this study, a total of 187 subjects who met the inclusion and exclusion criteria were enrolled, of which 74 patients refused the CPAP treatment plan but agreed to participate in the experiment, and 1 patients declined to undergo neurological function assessment during follow-up. Ultimately, 73 individuals were included in the control group. A total of 113 patients received the CPAP treatment plan and voluntarily joined the study, among which 12 patients could not tolerate the pressure titration on the first night. Adherence criteria were defined per current clinical practice guidelines as ≥12 nights of CPAP therapy with ≥4 h/night usage within a 14-day period (9). 32 subjects were excluded due to insufficient therapeutic adherence, one patients were excluded from data analysis because of missing or incomplete data on CPAP use, and 68 patients who met the criteria were ultimately included in the CPAP group. The overall CPAP adherence rate reached 67.3% (68/101) with a mean nightly usage duration of 5.82 ± 1.87 h. The detailed research flowchart is shown in Figure 1. Cross-group analysis of baseline characteristics revealed no statistically significant differences in demographic profiles, sleep-related questionnaire scores, and neurological assessments of stroke severity (all *p* ≥ 0.05; see Table 1), indicating comparability between the two groups.

**Table 1:** Demographic data and questionnaires between two groups.

Characteristics	CPAP (*n* = 68)	Control (*n* = 73)	*χ^2^/t/Z* value	*P* value
Male, *n* (%)	55 (80.9%)	49 (72.1%)	1.471	0.312
Age, years	58.37 ± 15.32	60.51 ± 10.98	−0.939	0.349
BMI, kg/m^2^	26.38 ± 3.62	26.38 ± 3.3	0.003	0.997
Smoking, *n* (%)	26 (38.2%)	31 (45.6%)	0.755	0.487
Drinking, *n* (%)	21 (30.9%)	27 (39.7%)	2.037	0.370
Hypertension status (%)	44 (64.7%)	51 (75%)	1.711	0.262
Diabetes status (%)	18 (26.5)	20 (29.4%)	0.146	0.849
CAD status (%)	6 (8.8%)	8 (11.8%)	0.119	0.730
AF	4 (5.9%)	5 (7.4%)	0.319	0.779
ESS	6 (3,12)	7 (3.25,12)	−0.268	0.788
PSQI	8 (4.25,11)	8 (5.25,11.75)	−0.587	0.557
HADS(A)	1.5 (0,3)	1 (0,2)	−1.071	0.284
HADS(D)	2 (1,3.75)	1.5 (0,4)	−1.045	0.296
SCD-Q9	2 (2,3)	2 (2,2)	−1.538	0.124
MoCA	20.28 ± 4.67	20.53 ± 5.94	0.273	0.786
RBD	3 (1,4)	2 (1,3)	−1.349	0.177
NIHSS	2 (0,3)	2 (1,3)	−0.821	0.412
mRS	2 (2,3)	2 (2,2)	−0.860	0.390
BI	78.38 ± 12.65	78.75 ± 14.54	−1.57	0.875

Data presented as mean ± SD (Independent *t*-test), median (IQR) (Mann–Whitney U test), or *n* (%) (Chi-squared test). BMI, body mass index; CAD, Coronary Artery Disease; AF, Atrial Fibrillation; ESS, Epworth Sleepiness Scale; PSOI, Pittsburgh Sleep Quality Index; SCD-Q9, Subjective Cognitive Decline Questionnaire-9; HADS, Hospital Anxiety and Depression Scale; MoCA, Montreal Cognitive Assessment Scale; RBD, REM Sleep Behavior Disorder Screening Questionnaire; NIHSS, National Institutes of Health Stroke Scale; mRS, Modified Rankin Scale; BI, Barthel Index. The measured data is expressed as the median (interquartile range) due to its skew distribution.

### 3.2 Analysis of neuroimaging characteristics

Comparisons of IS-related clinical features between groups are presented in Table 2, with no significant differences observed (all *p* ≥ 0.05).

**Table 2:** Comparison of imaging characteristics of lesions between the two groups.

Characteristics		CPAP (*n* = 68)	Control (*n* = 73)	*χ^2^/t/Z* value	*P* value
Laterality [number (%)]	Unilateral	19 (27.9%)	12 (17.6%)	2.047	0.220
	Bilateral	49 (72.1%)	56 (82.4%)		
Multiplicity [number (%)]	Isolated	25 (38.6%)	18 (26.5%)	1.666	0.268
	Multiple	43 (63.2%)	50 (73.5%)		
Number of stroke [number (%)]	≤4	47 (69.1%)	49 (72.1%)	0.142	0.851
	>4	21 (30.9%)	19 (27.9%)		
Site of IS [number (%)]	Pre-cycle	32 (47.1%)	35 (51.5%)	2.485	0.307
	post-cycle	16 (23.5%)	9 (13.2%)		
	Pre + post cycle	20 (29.4%)	24 (35.3%)		

IS, Ischemic Stroke.

### 3.3 Baseline PSG and sleep spindle parameters and CPAP intervention effects

As shown in Table 3, no baseline differences in PSG or sleep spindle parameters were detected between the groups. Post-CPAP treatment, sleep efficiency significantly improved (*p* = 0.002), accompanied by sleep architecture remodeling: a reduction in N1/N2 stage proportions and an increase in N3/REM stage proportions (all *p* < 0.05). Concomitant optimization occurred across respiratory metrics, including the arousal index, AHI, REM-AHI, NREM-AHI, ODI, mean SpO_2_, minimum oxygen saturation, and RERAI (all *p* < 0.05). Furthermore, CPAP increased N2-SSD, prolonged N2 sleep spindle duration, and increased both sleep spindle count and duration in N3 sleep.

**Table 3:** Comparison of sleep parameters: between groups and within CPAP group.

Polysomnography	Control	Pre-CPAP	Post-CPAP	*P*^a^ value	*P*^b^ value
TST (min)	390.76 ± 119.34	412.67 ± 106.02	420.99 ± 114.55	0.260	0.071
S-efficiency (%)	79.76 ± 12.60	78.34 ± 15.79	83.7 ± 12.88	0.561	0.002
SOL (min)	7.00 (3.15,12.38)	7.05 (3.15, 14.9)	5.8 (3.25, 13.7)	0.886	0.623
REM sleep latency (min)	98.25 (43.13, 144.63)	87.5 (42, 151.5)	81 (50.5, 129.5)	0.702	0.387
WASO (min)	73.95 (35.37, 111.3)	68.4 (37.33, 140.93)	50.05 (24.52, 89.92)	0.478	0.05
N1%	8.6 (4.73, 19.58)	6.95 (3.23, 14.83)	5 (2.13, 9.95)	0.112	0.023
N2%	60.05 (52.9, 66.53)	61.55 (52.18, 70.68)	54.95 (46.1, 66.28)	0.244	0.006
N3%	10.4 (7.1, 19.03)	9.9 (5.43, 16.68)	13.95 (7.8, 22.85)	0.191	<0.001
REM%	12.3 (7.83, 18)	14 (9.18, 20.5)	21.65 (14.9, 27.88)	0.251	<0.001
AHI	30.55 (22.5, 43.43)	34.45 (22.95, 52.55)	4.8 (2.72, 8.05)	0.448	<0.001
REM-AHI	31.2 (16.53, 49.86)	32.95 (18.78, 47.33)	4.35 (1.4, 8.23)	0.934	<0.001
NREM-AHI	30.1 (20.43, 43)	28.9 (20.55, 52.03)	3.85 (2.23, 8.3)	0.433	<0.001
ODI	34.75 (19.35, 53.05)	31.25 (19.3, 49.85)	6.45 (2.82, 11.85)	0.807	<0.001
Arousal Index	32.75 (21.35, 49.23)	31.7 (21.83, 46.63)	20.35 (11.45, 32.45)	0.606	<0.001
Mean SpO_2_ (%)	94.65 ± 2.01	93.83 ± 2.98	95.79 ± 1.29	0.081	<0.001
Min-SpO_2_ (%)	79.31 ± 9.52	80.88 ± 8.60	88.82 ± 4.76	0.314	<0.001
RERAI	5.8 (2.3, 12.38)	6.1 (3.4, 12.83)	0.4 (0.1, 1)	0.679	<0.001
N2-SS count	419 (242.5, 806)	513.5 (235.25, 722)	492.5 (242.25, 843)	0.951	0.375
N2-SSD	1.91 (1.14, 3.63)	1.95 (1.09, 2.84)	2.50 (1.41, 3.33)	0.411	0.036
N2-SS duration (s)	0.7 (0.6, 0.8)	0.7 (0.6, 0.8)	0.6 (0.6, 0.7)	0.674	<0.001
N2-SS max-amplitude (μV)	56 (51, 63.74)	53.5 (44, 64)	45 (40, 59)	0.126	0.052
N3-SS count	55 (25.5, 170.5)	42.5 (12, 173.5)	93.5 (24.75, 195.75)	0.321	0.048
N3-SSD	2.04 (0.94, 3.96)	1.61 (0.54,2.80)	1.54,(0.81, 2.61)	0.132	0.640
N3-SS duration (s)	0.65 (0.6, 0.8)	0.6 (0.6, 0.8)	0.6 (0.675, 0.875)	0.124	0.04
N3-SS max-amplitude (μV)	58 (49, 64)	53 (42, 66.75)	46.5 (41, 59.75)	0.466	0.089

TST, total sleep time; S-efficiency, sleep efficiency; SOL, sleep onset latency; WASO, wake time after sleep onset; N = NREM, non-rapid eye movement; REM, rapid eye movement sleep; AHI, apnoea-hypopnea index; ODl, oxygen desaturation index; Min-SpO_2_, minimum pulse oxygen saturation; RERAI, respiratory effort-related arousal index; N2-SS, N2 sleep spindle; N3-SS, N3 sleep spindle; SSD, sleep spindle density.

P^a^, The comparison of clinical data between the CPAP group and the Control group, if the data is normally distributed, two-sample *t*-test is used; if the data is not normally distributed, Mann–Whitney U test is used; P^b^, The comparison of parameters before and after treatment in the CPAP group, for data that is normally distributed, a paired *t*-test is used; for data that is not normally distributed, Wilcoxon rank-sum test is used.

### 3.4 30-day neurological outcome comparisons

**Figure 2:**
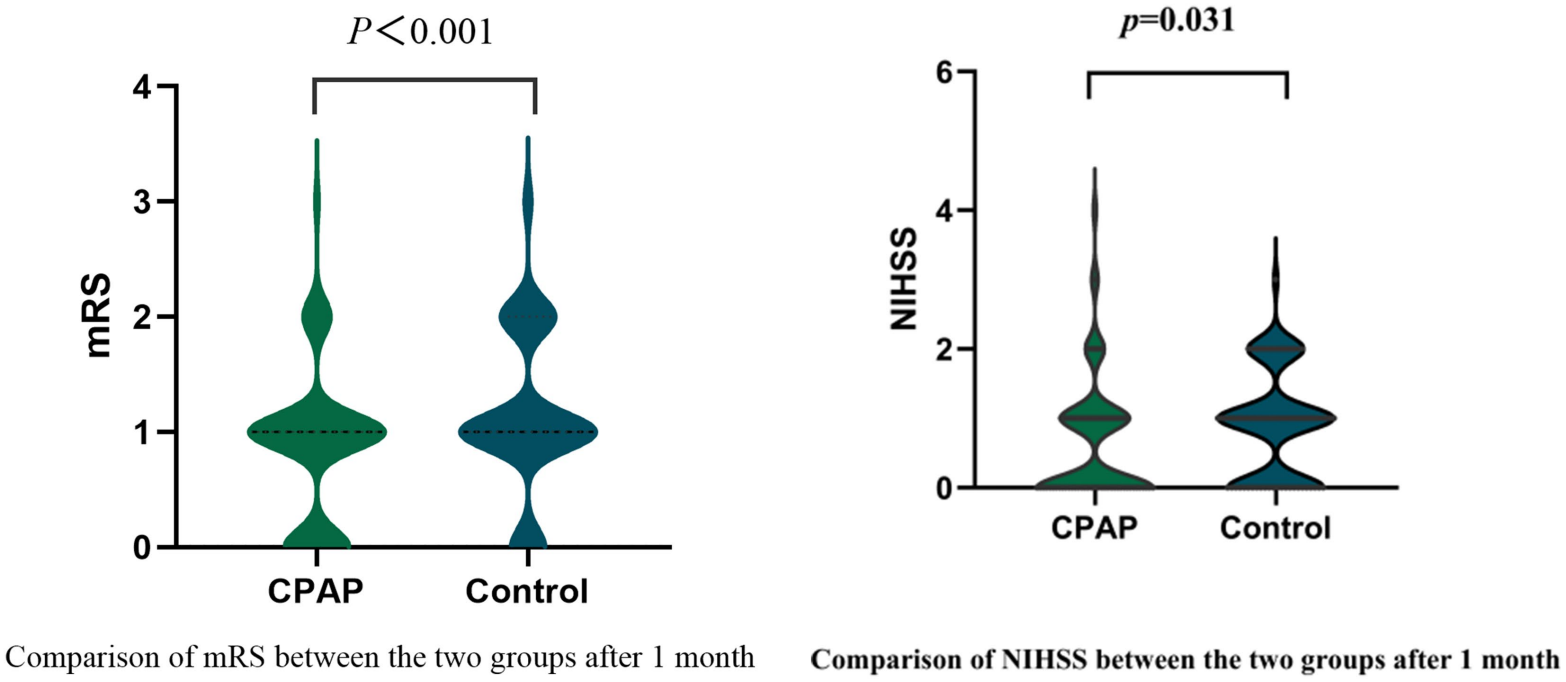
Comparison of mRS/NIHSS between the two groups after 1 month.

At the 30-day follow-up after stroke onset, the CPAP group demonstrated significantly lower mRS (*p* < 0.001) and NIHSS (*p* = 0.031) scores than did the control group, although BI scores were not significantly different between the groups (*p* = 0.086) (Table 4, Figure 2).

**Table 4:** Neurological function scores after CPAP treatment.

Characteristic	CPAP	Control	*χ*^2^/*t*/*Z* value	*p* value
BI	92.72 ± 8.39	89.71 ± 11.68	1.728	0.086
mRS	1 (0,1)	1 (1,1)	−4.214	<0.001
NIHSS	0 (0,1)	0 (1,1)	−2.157	0.031

### 3.5 Correlation analysis

Spearman correlation analysis (Table 5) revealed positive associations between the 30-day mRS score and the AHI (*r* = 0.242, *p* < 0.05), REM-AHI (*r* = 0.497, *p* < 0.05), and NREM-AHI (*r* = 0.275, *p* < 0.05). Negative correlations were observed with N3 sleep percentage (*r* = −0.363, *p* < 0.05), N2-SSD (*r* = −0.372, *p* < 0.05), and mean oxygen saturation (*r* = −0.336, *p* < 0.05).

**Table 5:** Correlation analysis of mRS scores with PSG parameters and spindles indicators.

Characteristics	*r*	*p*
N3%	−0.363*	0.03
REM-AHI	0.497**	<0.001
NREM-AHI	0.275*	0.023
AHI	0.242*	0.047
N2-SSD	−0.372**	0.002
Mean SpO_2_ (%)	−0.336**	0.005

*p < 0.05, **p < 0.01.

### 3.6 Ordered logistic regression analysis

**Figure 3:**
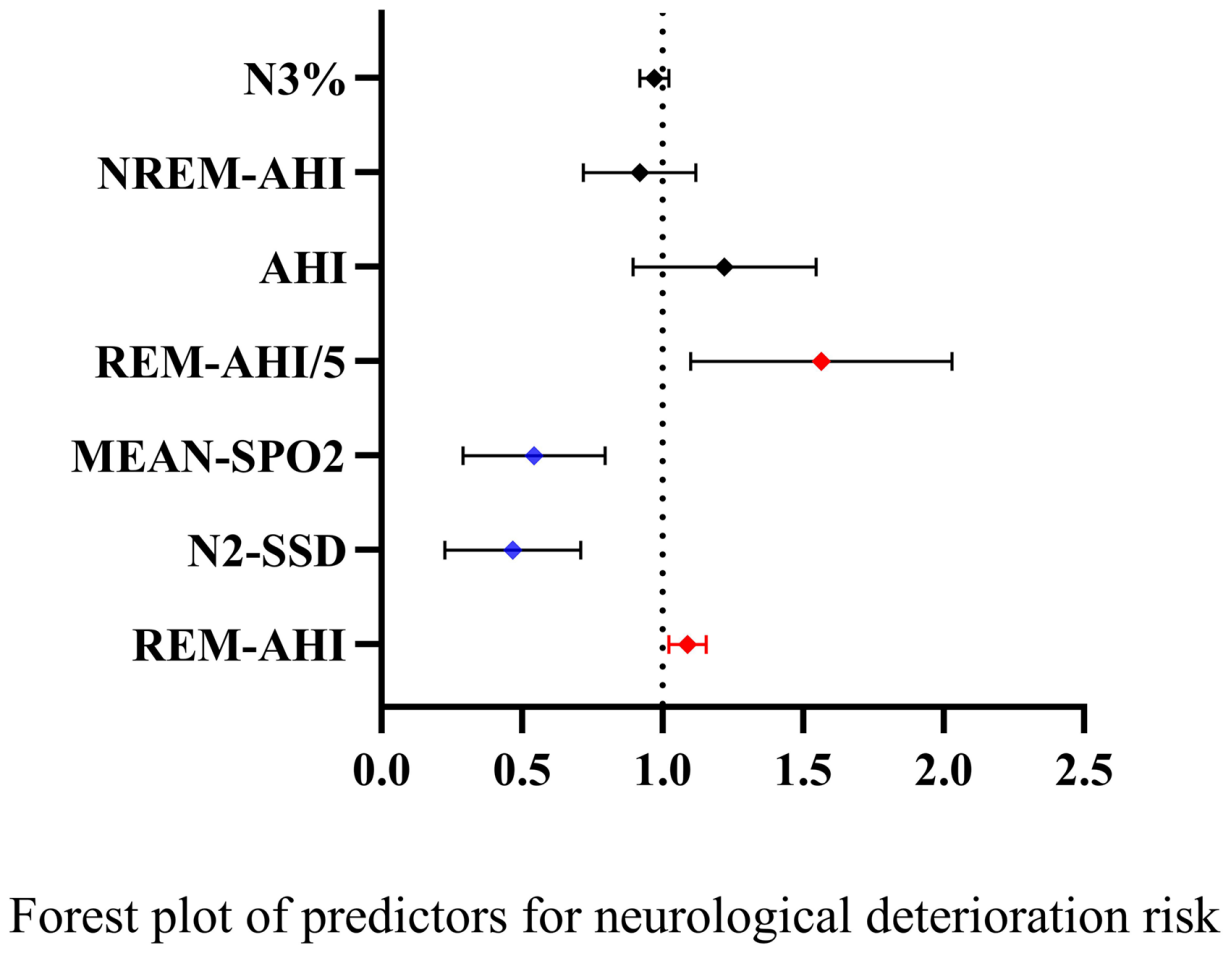
Forest plot of predictors for neurological deterioration risk.

Variables showing significant differences post-CPAP were included in an ordered logistic regression model with the 30-day mRS score as the dependent variable. REM-AHI, N2-SSD, and mean oxygen saturation emerged as significant independent predictors of mRS scores (all *p* < 0.05; Table 6; Figure 3). For more intuitive clinical interpretation, we analyzed REM-AHI in 5-unit increments (REM-AHI/5). For every 1% increase in mean oxygen saturation, patients’ risk of neurologic deterioration was reduced by approximately 50% (OR = 0.504, 95% CI: 0.312–0.831). Similarly, for every 1-unit increase in N2-SSD, the risk was reduced by approximately 57% (OR = 0.426, 95% CI: 0.249–0.727). In contrast, for every 1-unit increase in REM-AHI, patients’ risk of neurologic deterioration increased by approximately 8.7% (OR = 1.087, 95% CI: 1.024–1.156); when REM-AHI increased by 5 units, the risk increased by 52% (OR = 1.52, 95% CI: 1.125–2.052). In contrast, NREM-AHI, AHI, and N3% were not significantly correlated with neurologic prognosis (all *p* > 0.05).

**Table 6:** Ordered logistic regression of variables related to mRS.

Variables	*β* Coefficient	SE	*p*-Value	OR	95%CI
REM-AHI	0.084	0.031	0.006	1.087	(1.024, 1.156)
NREM-AHI	−0.095	0.116	0.412	0.909	(0.724, 1.124)
AHI	0.176	0.137	0.198	1.192	(0.912, 1.560)
MEAN-SPO_2_	−0.685	0.244	0.005	0.504	(0.312, 0.813)
N2-SSD	−0.854	0.273	0.002	0.426	(0.249, 0.727)
N3%	−0.030	0.027	0.272	0.970	(0.920, 1.023)
REM-AHI/5	0.419	0.153	0.006	1.520	(1.125, 2.052)

## 4 Discussion

Guided by the Expert Consensus on Obstructive Sleep Apnea and Stroke Management (22), this study initiated 14-day CPAP treatment within 48 h of stroke onset in 68 patients with IS complicated by moderate-to-severe OSA. By comparing our findings with those of untreated controls, we validated the clinical value of early therapeutic intervention. Among the clinical parameters demonstrating positive responses to CPAP, REM-AHI, N2-SSD, and mean SpO_2_ may be critical predictors of neurological outcomes, which provides a mechanistic basis for CPAP efficacy in prognosis optimization.

The association between mean SpO_2_ and neurological outcomes is biologically plausible, given prior evidence elucidating the negative association between sleep-related hypoxia and neurological recovery, along with its underlying pathophysiological mechanisms (23, 24). Notably, REM-AHI and N2-SSD were correlated with neurological prognosis. Previous studies have established that apnea-hypopnea events that occur during REM sleep stage are associated with more severe clinical consequences for patients (25, 26). The results of a previous study by our group also revealed that patients with predominantly REM-OSA (rapid eye movement-related obstructive sleep apnea) have a worse prognosis than do ischemic stroke patients with NREM-OSA (nonrapid eye movement-related obstructive sleep apnea). OSA-induced cerebral hypoxia and hemodynamic fluctuations may exacerbate injury in peri-ischemic penumbral regions (4). The duration of apnea events, modulated by chemoreflex drive and arousal thresholds (27), is prolonged during REM sleep because of diminished respiratory effort and delayed cortical arousal. Furthermore, reduced noradrenergic activity in the locus coeruleus during REM stage suppresses both upper airway dilator muscles and respiratory drive (26). Therefore, OSA may further deteriorate in REM and exacerbate the harm caused to stroke patients. This study also revealed that the REM-AHI affects the prognosis of patients. That is, the higher the REM-AHI is, the worse the short-term prognostic outcomes for patients may be.

The study protocol excluded patients with suboptimal CPAP adherence. These patients initiated therapy within 48 h poststroke onset, which yielded favorable short-term respiratory compliance. While optimal CPAP adherence is clinically critical for targeting REM-related respiratory event suppression (28), longitudinal data reveal persistent challenges in sustaining therapeutic engagement. A retrospective study revealed that the CPAP usage rates in stroke patients after treatment were 58, 53, 48, 45, and 39% at 3, 6, 12, 24, and 60 months, respectively (29). Moreover, the predominance of circadian-driven REM sleep during early morning hours necessitates extended nocturnal usage protocols to achieve optimal therapeutic coverage. Encouragingly, a growing number of clinical studies are actively seeking means to optimize adherence with good results, and beliefs about behavioral change and telemonitoring follow-up may be possible answers (30–32), with future research directed toward combining theory-driven behavioral approaches with telemedicine technology to improve real-world CPAP adherence rates. Patients with more severe OSA generally have poorer cerebral responsiveness to hypoxia because of the moderate-strength relationship between the cerebral blood flow response to hypoxia and the AHI and nocturnal oxyhemoglobin saturation. One study reported the greatest change in cerebral blood flow response to hypoxia after CPAP treatment in patients with the highest AHI (33), which suggests that patients with severe OSA may benefit the most from CPAP treatment. In our study, we did not assess the prognosis by subdividing the severity of the patients, and we further investigated this point at a later stage. At the same time, to ensure the generalizability and clinical translational value of the findings, future multicenter validation studies in different populations are still needed.

According to the AASM normal ranges for sleep stage percentages, the sleep architecture of patients with OSA was disturbed, exhibiting a higher percentage of N1 and N2 stage and a decrease in N3 and REM stage. Following short-term CPAP intervention, the sleep structure of the patients was improved, with an increase in the percentage of deep sleep, a rebound in REM sleep, and a decrease in the RERAI observed. These changes directly ameliorated hypoxemia and sleep fragmentation. RERAI plays a bidirectional regulatory identity in neuromodulation, on the one hand, it can terminate life-threatening apnea, on the other hand, excessive arousals lead to sleep structure disruption, impaired neuronal repair, interference with sleep spindles formation, and ultimately cause deterioration of neurological function.

Sleep spindles are burst signals in the EEG of the mammalian brain during sleep and are electrophysiological surface correlates of thalamic neuronal oscillations with an amplitude of 11–16 Hz (34). Spindles are generated by coordinated activity between the thalamus, the thalamic reticular nucleus, and the neocortex (thalamocortical loops). Spindle activity prevents arousing stimuli from reaching the cortex and thereby prevents awakenings during NREM sleep. Sleep spindle activity is involved in several processes, such as sleep architecture, sensory processing, synaptic plasticity, memory formation and cognitive ability. Sleep spindle density is closely related to cognitive functions such as resistance to external disturbances during sleep, individual intelligence and memory consolidation (35). Previous studies have shown that the intensity and consistency of spindle wave activity are significantly reduced in stroke patients (36, 37), and that brain damage affects the generation of sleep spindle wave activity. A number of rodent experiments and clinical studies have preliminarily demonstrated that cognitive impairments such as memory and other cognitive functions induced by stroke are associated with sleep spindles (38, 39).

In the present study, we found that CPAP treatment increased N2-SSD, a result similar to previous studies (40, 41), which may be a biomarker of treatment effectiveness. However, the increase in SSD was not manifested in N3 stage. The occurrence of spindle waves is dependent on the level of hyperpolarization in the thalamocortical network, and their frequency is influenced by the duration of the hyperpolarized rebound sequence of thalamocortical cells (42). If the rebound sequence is long, the spindle wave slows down. In general, spindle waves are abundant and fast in light sleep and few and slow in deep sleep (43, 44). However, the percentage of N3 sleep is significantly reduced in OSA patients, which implies that the hyperpolarization of the thalamocortex in these patients is maintained at a moderate level. After treatment with CPAP, patients achieve slow-wave sleep with an increase in the negative membrane potential of the thalamocortical network along with the optimization of the sleep structure, which may explain why the N3-SSD did not significantly change. The basic principle of CPAP treatment is to generate gas of a certain pressure by means of a ventilator, which creates airborne scaffolding in the patients’ upper airway to form a gaseous scaffolding to support the collapsed area and keep the upper airway open during sleep, which allows for the correction of hypoxia in the cells within the CNS and thalamus without apnea hypo-potentials; therefore, these cells are more capable of generating sleep spindle waves, which in turn allows the patient to show greater potential for a good prognosis. Increased sleep spindle wave activity and coherence from the acute to the chronic phase of stroke suggest a reversible mechanism allowing for the possibility of spindle wave recovery (45).

However, this study did not identify N3-SSD as a predictor of short term neurological prognosis. Currently, there are fewer studies on the N3-SSD because sleep spindles are a characteristic electrophysiological manifestation of N2 stage and appear less frequently in N3 stage. Moreover, the sleep structure of patients with OSA combined with ischemic stroke is disrupted, and there is a significant reduction in N3 stage sleep, which is dominated by slow-wave activity, and its neural oscillatory pattern, neurochemical environment, and potential functions are significantly different from those of N2 sleep. The incidence of sleep spindle in N3 stage, their morphological characteristics and their coupling with slow-wave activity may also be different from N2 stage. Therefore, impairment of N3-SSD after stroke might reflect a different mechanism of neurologic injury or recovery pathway than N2-SSD impairment, or its predictive value for functional recovery might be less sensitive than that of N2-SSD. Future studies should conduct in-depth investigations into how stroke specifically affects spindle activity across different sleep stages. Additionally, incorporating neuroimaging and molecular marker studies would help elucidate the unique or synergistic mechanisms of N2-SSD and N3-SSD in post-stroke neural repair and recovery.

Studies published as early as 1960s that reported sleep spindle as a favorable prognostic indicator for patients surviving coma, stroke, encephalopathy, and traumatic brain injury (46) seem to have been ignored. In recent years, it has also been increasingly suggested that N2 stage biomarkers can be used as predictors of prognosis in critical illness patients (47, 48). The results of this study revealed that the N2-SSD is an independent risk factor affecting the prognosis of patients with OSA-IS. Intervention with spindles during sleep is expected to promote neuromodulation and perhaps improve subsequent prognosis and stroke rehabilitation, and sleep spindle may become a new therapeutic target.

The primary endpoint of this study was neurologic recovery at 30 days after stroke, an early time window of clinical importance that effectively reflects the response to acute-phase interventions and the potential for early recovery (49). However, we recognize that a 30-day assessment is insufficient to fully depict the long-term neuroplasticity process after stroke, the eventual plateau period of functional recovery, or the risk of stroke recurrence. Although long-term outcomes were not tracked in this study, on the basis of the potential mechanisms of sleep spindles (especially N2-SSD) in neuroprotection, synaptic plasticity, and brain network reorganization (35), acutely impaired N2-SSD may not only correlate with short-term recovery but also be predictive of long-term neurological prognosis. Similarly, whether the persistence of REM-AHI is associated with an increased risk of long-term cardiovascular events or stroke recurrence also deserves to be explored in depth. Therefore, future studies should aim to extend the follow-up period and systematically assess the value of N2-SSD and REM-AHI in predicting long-term neurologic outcomes, cognitive function, quality of life, and risk of stroke recurrence in stroke patients.

REM-AHI and N2-SSD modulate neuroplasticity via independent mechanisms. CPAP treatment selectively improves the abnormal activity of these two biomarkers, thereby mediating their positive impact on patients’ neurological prognosis. The identification of novel electrophysiological markers, such as REM-AHI and N2-SSD, is expected to overcome the limitations of traditional AHI metrics in predicting CPAP efficacy.

The limitations of this study are the relatively small sample size, which may limit the generalizability of the results. The lack of long-term follow-up data is also a notable limitation, which prevents us from drawing comprehensive conclusions about the lasting effects of CPAP treatment. Although we observed significant improvements in the short term, further studies are needed to verify whether these results carry over into the long term. This study was conducted in patients with moderate to severe OSA who were able to voluntarily accept and cooperate with CPAP treatment. Although the sample size was sufficient to detect significant effects for primary outcomes, it remained limited for multivariate modeling and subgroup analyses. This selective inclusion may introduce bias into the research findings, and the conclusions may only be generalizable to patient populations with good adherence. In addition, this study was a single-center design, and population characteristics may affect the extrapolation of the results. Therefore, larger-scale multicenter prospective cohort studies in diverse populations remain needed to validate the clinical applicability of these findings.

## 5 Conclusion

Our study demonstrated that initiating CPAP therapy within 48 h after stroke significantly improved short-term neuro-prognostic function in patients with IS combined with moderate-to-severe OSA. Clinical strategies should be optimized by controlling the timing of CPAP initiation and maximizing respiratory therapy compliance. REM-AHI, N2-SSD and mean SpO_2_ are closely related to prognosis. The application of these prognostic indicators in clinical practice will be an important focus of our future research. Given the substantial burden of vascular morbidity and mortality in stroke patients with OSA, exploring novel therapeutic approaches to improve patient prognosis is imperative.

## Data Availability

The raw data supporting the conclusions of this article will be made available by the authors, without undue reservation.
